# The Impact of Dietary Curcumin on the Growth Performance, Intestinal Antibacterial Capacity, and Haemato-Biochemical Parameters of Gilthead Seabream (*Sparus aurata*)

**DOI:** 10.3390/ani11061779

**Published:** 2021-06-15

**Authors:** Ahmed M. Ashry, Aziza M. Hassan, Mahmoud M. Habiba, Ahmed El-Zayat, Mohamed E. El-Sharnouby, Hani Sewilam, Mahmoud A.O. Dawood

**Affiliations:** 1 National Institute of Oceanography and Fisheries, NIOF, Cairo 11865, Egypt; ahmed_ashry74@yahoo.com (A.M.A.); mahmoudhabiba40@yahoo.com (M.M.H.); 2 Department of Biotechnology, College of Science, Taif University, P.O. Box 11099, Taif 21944, Saudi Arabia; a.hasn@tu.edu.sa (A.M.H.); m.sharnouby@tu.edu.sa (M.E.E.-S.); 3 Department of Animal Production, Faculty of Agriculture, Al-Azhar University, Nasr City, Cairo 11651, Egypt; Ahmedelzayat.5@azhar.edu.eg; 4 The Center for Applied Research on the Environment and Sustainability, The American University in Cairo, Cairo 11835, Egypt; sewilam@aucegypt.edu; 5 Department of Engineering Hydrology, RWTH Aachen University, 52062 Aachen, Germany; 6 Department of Animal Production, Faculty of Agriculture, Kafrelsheikh University, Kafrelsheikh 33512, Egypt

**Keywords:** *Curcuma longa*, seabream, production, antibacterial, health status

## Abstract

**Simple Summary:**

In aquaculture, dietary curcumin has been shown to enhance the growth rate, antioxidative status, immunity, and disease resistance of several finfish species. Nevertheless, the potential role of curcumin has not been evaluated in Gilthead seabream as yet. Herein, we tested the effect of dietary curcumin on the growth performance, intestinal antibacterial capacity, and haemato-biochemical parameters of Gilthead seabream. Curcumin was mixed with the basal diet at rates of 0, 1.5, 2, 2.5, and 3%, then fed to the fish for 150 days. The results indicated marked improvements in the growth performance, feed efficiency, and antibacterial capacity of the fish. Further, curcumin enhanced the hematological indices and regulated the biochemical blood metabolites of Gilthead seabream. Dietary curcumin is recommended at a rate of 2–3% to improve the performance of Gilthead seabream.

**Abstract:**

The need to replace antibiotics in aquafeed is increasing, and alternative safe substances are now encouraged for sustainable aquaculture activity. Curcumin is regarded as a multifunctional feed additive with growth-promoting and immunostimulant potential. Thus, this study evaluated dietary inclusion of curcumin at rates of 0, 1.5, 2, 2.5, and 3% in the diets of Gilthead seabream for 150 days. The results showed an improved final body weight, weight gain, specific growth rate, and feed conversion ratio in fish treated with curcumin, in a dose-dependent manner. The highest growth performance was observed in fish fed a diet supplemented with 3% curcumin. The results also showed lowered activity of pathogenic bacteria (*Vibrio* spp. and *Faecal coliform*) in the intestines of Gilthead seabream fed a diet with curcumin inclusion, in a dose-dependent manner. The hematological indices were within the normal range for healthy fish, without meaningful effects except for hematocrit, hemoglobin, red blood cells (RBCs), and white blood cells (WBCs), which were markedly increased by dietary curcumin. Phagocytic activity was obviously enhanced by dietary curcumin, compared with the control. The biochemical blood metabolites related to liver function (alkaline phosphatase (ALP), aspartate aminotransferase (AST), alanine aminotransferase (ALT)), renal tissue (urea), and total cholesterol were within the normal values, without significant differences. Overall, the inclusion of curcumin at a rate of 2–3% improved the growth performance and well-being of Gilthead seabream.

## 1. Introduction

The aquaculture industry is known for its contribution to food safety and meeting the animal protein needs of humanity [1]. Thus, farmers are now actively applying intensive and semi-intensive aquaculture systems to meet the increased demand for aquatic animals [2,3]. Although intensive systems result in high production at the lowest possible cost, they have adverse impacts on the aquatic organisms’ performance and well-being [4]. Intensive systems result in stress and immunosuppression, leading to higher mortality and substantial economic losses [5]. Antibiotics and chemotherapy have been successfully applied to reduce the impacts of intensive conditions on the performance of aquatic animals [6]. However, the continuous usage of antibiotics has had several negative consequences [7,8]. Indeed, the accumulated derivatives of antibiotics have led to weak natural immunity of aquatic animals, and the growth of antibiotic-resistant bacterial strains [9]. Antibiotics can also be indirectly transmitted to the human body, resulting in hazardous impacts [10]. Nutraceuticals are an alternative now being recommended for sustainable fish farming [11,12,13].

Turmeric (*Curcuma longa* Linn.) is a functional spicy herbal plant affiliated with the Zingiberaceae family, which was initially grown in tropical areas [14,15]. In some Asian countries, turmeric is called “golden spice” and is regarded as an essential herb for cooking and health [16]. Curcumin is a polyphenolic content extracted from the turmeric herb, and has several pharmaceutical effects [17,18]. Markedly, curcumin has growth-promoting, antibacterial, immunostimulant, antioxidative, and anti-inflammation properties in humans and animals [19,20]. The flavor of curcumin also enhances the palatability of food and feed [21]. In aquaculture, dietary curcumin has been shown to enhance the growth rate, antioxidative status, immunity, and disease resistance of several finfish species [18,22]. However, the potential role of curcumin has not been evaluated in Gilthead seabream (*Sparus aurata*) as yet.

The Gilthead seabream belongs to the Sparidae family, which originated in the Mediterranean Sea area. In this area, several countries (e.g., Greece, Turkey, Spain, Tunisia, and Egypt) have recently begun farming Gilthead seabream in intensive systems, due to high demand and its commercial value [23,24]. The inclusion of curcumin in Gilthead seabream diets is likely to be a practical solution to enhance their productivity and health [25]. Thus, this study assessed the potential of curcumin to enhance the growth rate, intestinal antibacterial capacity, and blood health of Gilthead seabream.

## 2. Materials and Methods

### 2.1. Test Diets

The basal test diet was prepared containing 44.11% protein and 15.21% lipids, using fish meal, shrimp meal, soybean meal, yellow corn, corn gluten, wheat middling, dicalcium phosphate, vitamin-mineral mixture, and fish oil (Table 1). Then, all of the dry ingredients were mixed and divided into five portions by mixing the powdered ingredients. Curcumin was mixed with the fish oil at the rates of 0, 1.5, 2, 2.5, and 3%, then included in the test diets. Powdered curcumin was obtained from a local market, and confirmed by checking the color and flavor before mixing with the remaining ingredients. Water was added to the mixed formulation to produce pellets of dough (2–3 mm) using a lab meat mincer fixed with a pelletizer (El-Adl Co.^TM^, Tanta, Egypt). The pelleted diets were then dried in the oven (Memmert UN110, Buchenbach, Germany) at 50 °C for two hours, before being stored in a freezer in airtight bags until use.

### 2.2. Experimental Design

A stock of Gilthead seabream (*Sparus aurata*) fingerlings was obtained from the National Institute of Oceanography and Fisheries (NIOF), Alexandria, Egypt, and introduced to concrete tanks of 1 × 8 × 3 m (L × W × D). Twenty homogeneous fish were collected from the stock and washed with fresh water, then kept in a plastic bag until death before being frozen at −20 °C for the assessment of initial carcass composition. Each tank was fixed with inlet and outlet water sources with continuous aeration. The fish were adapted to the laboratory conditions for ten days and hand fed the basal diet three times daily (9:00, 12:00, and 15:00). Then, the fish were assigned to 15 hapas (1 × 1 × 1 m), and placed in five concrete tanks (1 × 8 × 3 m; L × W × D) with three hapas in each tank. Each hapa was stocked with 15 fish of an average initial weight of 20.00 ± 0.37 g/fish. Five groups of fish in triplicate were fed the test diets with curcumin at rates of 0, 1.5, 2, 2.5, and 3% for 150 days, three times daily (9:00, 12:00, and 15:00), by hand. The fish were fed the test diets to satiation, and the amount of feed was recorded for the calculation of feed intake. The weight of the fish was checked biweekly to obtain the growth rate and check the health status. The tanks were provided with an underground water source in a flow-through system, with a flow rate of 2 L/min. Water quality was checked regularly during the trial. The water quality values were as follows: salinity (32 ± 0.52 ppt), dissolved oxygen (5.32 ± 0.34 mg/L), water temperature (27.06 ± 0.32 °C), ammonia (0.02 ± 0.001 mg/L), and pH (7.1 ± 0.21).

### 2.3. Final Sampling

After 150 days, all fish were fasted for 24 h before the final sampling. Then, the fish were weighed and counted to calculate the growth-related indices using the following equations:

Weight gain (WG, g) = FBW − IBW
(1)

Specific growth rate (SGR, % day) = ((ln(FBW) − ln(IBW)) ÷ t (150 days)) × 100
(2)

Survival (%) = 100 × (FN ÷ IN)
(3)

where the initial (IBW) and final body weights (FBW) (g) of the fish, respectively; t is the duration of the experiment in days; and IN and FN are the initial and final number of fish, respectively.

Feed conversion ratio (FCR) = weight of feed (g) ÷ live weight gain (g)
(4)

Protein efficiency ratio (PER) = live weight gain (g) ÷ dry protein intake (g)
(5)

Then, five fish were randomly selected from each hapa for carcass chemical analysis. The collected fish were washed with freshwater, weighed, and kept at −20 °C. The fish were then dried and gently crushed into powder form. The diets and the fish whole body were analyzed for moisture, crude protein, crude lipids, and ash in triplicate, using standard methods [26]. In brief, the moisture content was evaluated following oven drying (Memmert UN110, Buchenbach, Germany) at 105 °C until a constant dry weight was reached. The ash content was detected using a muffle furnace (Heraeus Instruments K1252, Hanau, Germany) at 550 °C for 6 h. Crude protein was analyzed using the Micro-Kjeldahl apparatus (Foss Kjeltec 2200, Hillerqd, Denmark). Total lipid content was determined by petroleum ether extraction in the Soxhlet apparatus for 16 h.

### 2.4. Blood Sampling and Dissection

Another three fish per hapa were gently bled from the caudal vein, using 2.5 mL heparinized syringes to collect blood for hematological analysis. Using non-heparinized syringes, blood was also collected for serum separation. The samples were left for 4 h at 4 °C, then centrifuged at 3000× *g* for 15 min at 4 °C for serum collection. The serum samples were kept at −80 °C for further biochemical analysis. Then, the fish were dissected to separate the liver and viscera for measurement of the viscerasomatic index (VSI) and hepatosomatic index (HSI), using the following equations.

VSI (%) = weight of viscera ÷ weight of fish × 100
(6)

HSI (%) = weight of liver ÷ weight of fish × 100
(7)

### 2.5. Blood Analysis

The white blood cell (WBC) and red blood cell (RBC) counts, and hemoglobin concentration (Hb), were undertaken following standard procedures [27]. Hematocrit (Hct) was determined by the micro hematocrit method, while the hemoglobin (Hb) concentration was determined with a spectrophotometer (Model RA 1000, Technicon Corporation, Pittsburgh, PA, USA) at 540 nm, using the Blaxhall and Daisley [28] method. The monocyte, lymphocyte, and neutrophil differential counts were determined using the Wright Giemsa staining method. The mean corpuscular hemoglobin (MCH), mean corpuscular volume (MCV), and mean corpuscular hemoglobin concentration (MCHC) were calculated using the following formula [29]:

MCH (pg Hb/erythrocyte) = 10 × Hb ÷ RBCs
(8)

MCV (cm^3^/erythrocyte) = 10 × Hct ÷ RBCs (9)

MCHC (g Hb/100 mL erythrocytes) = 100 × Hb ÷ Hct
(10)

Serum total proteins and albumins were determined according to Doumas et al. [30] and Dumas [31]. Alanine aminotransferase (ALT) and aspartate aminotransferase (AST) activities were detected by following the method of Reitman and Frankel [32]. Alkaline phosphatase (ALP) enzyme activity was determined using commercially supplied kits by Pasteur Lab (Diagnostics Pasteur, Marnes la Coquette, France) [33]. Serum total cholesterol levels were estimated spectrophotometrically (RA-50 chemistry analyzer (DIAGNOSTICSMANUFACTURING LIMITED, BAYER, DUBLIN, IRELAND) by following the method of Schettler et al. [34]).

The leukocyte phagocytic function was determined following the method of Cai et al. [35]. The number of leukocytes that engulfed bacteria was counted as a percentage in relation to the total leukocyte number in the smear from the phagocytosis assay. By following Kawahara et al. [36], the phagocytic activity and phagocytic index were determined.

### 2.6. Intestinal Microbial Analysis

After blood sampling, the fish were dissected, and the distal intestine was carefully aseptically excised from each fish specimen (using sterile tools) and homogenized in 10 mL of 3% sterile sodium chloride solution. Concisely, ten-time dilutions of the stock samples were carried out to obtain serially diluted samples from 10^−1^ to 10^−5^. The bacteria population was determined by assays of growth on plated selective agar media, by taking one milliliter from the last dilution. The total count of viable bacteria (TBC) was determined by using seawater agar [37], whereas *Vibrio* spp. were determined by using thiosulfate-citrate-bile salt-sucrose (TCBS) agar [38]. For *Escherichia coli*, modified fecal coliform (mFC) agar was used (ISO (International Organization for Standardization) No. 9308/1, 1990). Incubation of the plates was carried out at 30 °C for 24–48 h for enumeration, except for the mFC medium, which was incubated at 44 °C for 24 h. de Man, Rogosa, and Sharpe (MRS) medium was used to cultivate the fermentative acid bacteria, which were incubated at 37 °C for 48 h under anaerobic conditions [39]. The tools were cleaned and sterilized between individual fish specimens. All samples were in triplicate for analysis.

### 2.7. Statistical Analysis

Shapiro–Wilk and Levene tests confirmed a normal distribution and homogeneity of variance. The obtained data were subjected to one-way ANOVA. Differences between means were tested at the *p* < 0.05 level using the Duncan test as a post-doc test. All the statistical analyses were carried out via SPSS version 22 (SPSS Inc., Armonk, NY, USA). The analyzed data are represented as the mean ± standard error (SE) (*n* = 3).

## 3. Results

### 3.1. Growth Performance

The final body weight (FBW) and weight gain (WG) were meaningfully increased by dietary curcumin in a dose dependent manner (Table 2). Fish fed 3% curcumin had the highest FBW (126.11 ± 0.38; *n* = 3) and WG (106.36 ± 0.48; *n* = 3), and fish fed a curcumin-free diet had the lowest FBW (112.56 ± 0.65; *n* = 3) and WG (92.38 ± 0.88; *n* = 3) (*p* < 0.05). Fish fed curcumin at 3% had a higher specific growth rate than fish fed the 0, 1.5, 2, and 2.5% levels of curcumin (*p* < 0.05). The highest feed conversion ratio (FCR) was observed in fish delivered 1.5% curcumin (1.72 ± 0.01; *n* = 3), while the lowest FCR value was seen in fish treated with 3% (1.35 ± 0.01; *n* = 3) (*p* < 0.05). Fish fed 0 and 2% had markedly lower FCR than fish fed 1.5%, and higher FCR than fish fed 2.5% (Table 2). The protein efficiency ratio (PER) was meaningfully increased by 2.5 and 3% additions of curcumin, compared to the 1.5 and 2% levels (*p* < 0.05). Further, fish treated with 3% had a higher PER than fish treated with 2.5% (Table 2). No marked effect of curcumin was shown on the survival rate of Gilthead seabream (*p* > 0.05).

### 3.2. Carcass Composition and Somatic Indices

The carcass composition of the initial samples showed a protein content of 57.28%, lipid content of 25.78%, and ash content of 15.80% (Table 3). After the feeding trial, the carcass composition showed insignificant differences in the dry matter, protein, lipid, and ash contents in Gilthead seabream treated with varying levels of curcumin (*p* > 0.05) (Table 3). The HSI and VSI were also not impacted by dietary curcumin (*p* > 0.05) (Table 3).

### 3.3. Intestinal Microbial Populations

**Figure 1:**
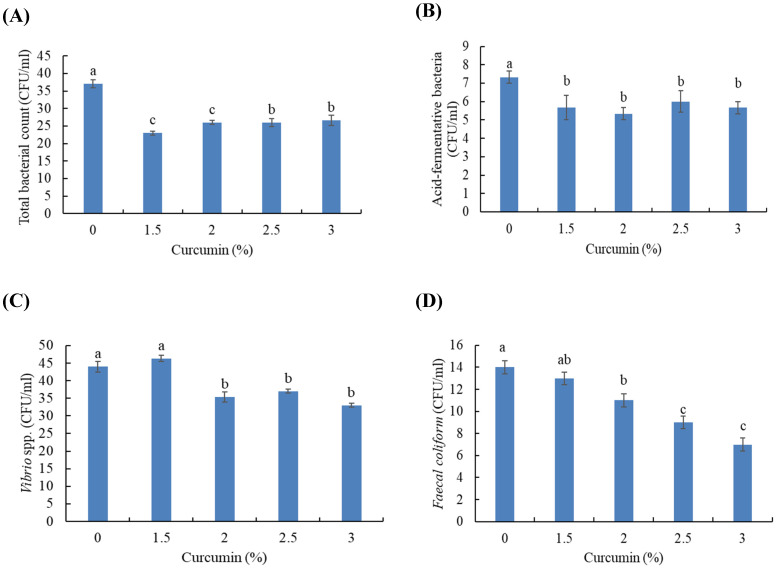
Intestinal microbial population in Gilthead seabream fed varying levels of dietary curcumin for 150 days: (**A**) total antibacterial count, (**B**) acid-fermentative bacteria, (**C**) *Vibrio* spp., and (**D**) *Faecal coliform*. A colony-forming unit (CFU) was used to check the count of bacteria per ml. Bars with different letters are significantly different (*p* < 0.05) (*n* = 3).

The total bacterial count (TBC) and acid-fermentative bacteria populations were lower in fish treated with curcumin than fish fed a curcumin-free diet (*p* < 0.05) (Figure 1A,B). Further, fish treated with the 1.5 and 2% levels had a lower TBC than fish fed 2.5 and 3%. The count of *Vibrio* spp. was lower in fish treated with 2.5 and 3% curcumin than fish treated with 0, 1.5, and 2% (*p* < 0.05) (Figure 1C). Fish fed curcumin at 2, 2.5, and 3% levels had a lower *Faecal coliform* count than fish fed the 0 and 1.5% levels, while fish fed 2.5 and 3% had lower *Faecal coliform* levels than fish fed 2% curcumin (*p* < 0.05) (Figure 1D). 

### 3.4. Hematological and Biochemical Indices

Hemoglobin levels, hematocrit, and WBCs were meaningfully higher in fish treated with curcumin than in fish fed curcumin-free diets (*p* < 0.05) (Table 4), while RBCs were higher in fish treated with curcumin at the 2, 2.5, and 3% levels than in fish treated with the 0 and 1.5% levels (*p* < 0.05). Additionally, the ALP, AST, ALT, urea, albumin, and total cholesterol were not meaningfully affected by dietary curcumin (*p* > 0.05) (Table 5).

### 3.5. Blood Immunity

**Figure 2:**
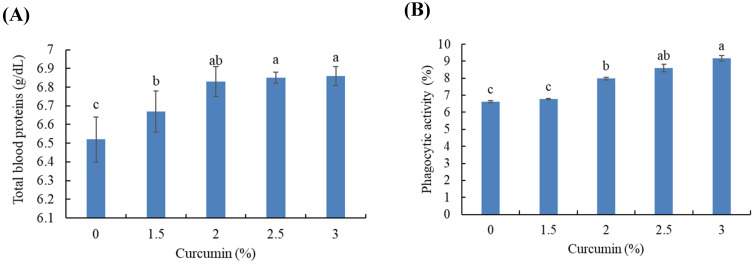
Blood total protein (**A**) and phagocytic activity (**B**) in Gilthead seabream fed varying levels of dietary curcumin for 150 days. Bars with different letters are significantly different (*p* < 0.05) (*n* = 3).

Blood total proteins were markedly increased in fish treated with curcumin, and fish fed the 2.5 and 3% levels had higher total protein than fish fed the 1.5% level (*p* < 0.05) (Figure 2A). Additionally, the phagocytic activity was meaningfully higher in fish fed 2, 2.5, and 3% curcumin than in fish fed 0 and 1.5% (*p* < 0.05) (Figure 2B).

## 4. Discussion

Using natural nutraceuticals in aquafeed is a key strategy for aquaculture sustainability [40,41,42]. Several medicinal herbs have been applied in aquaculture, and validated as growth-promoting and immunostimulant agents [43,44,45,46]. The results have suggested that medicinal supplements need to be investigated in a species-specific manner. Dietary curcumin has several beneficial effects on the growth performance, metabolic and physiological functions, immunity, antioxidative capacity, and disease resistance of finfish species [18,22]. Thus, including curcumin is highly recommended to enhance the productivity and wellbeing of Gilthead seabream. The results showed marked improvements in the growth rate, SGR, and FCR of Gilthead seabream fed curcumin, consistent with the findings of several previous studies. Including curcumin has resulted in enhanced growth performance in Nile tilapia (*Oreochromis niloticus*) [47], common carp (*Common carpio*) [48], grass carps (*Ctenopharyngodon idells*) [49], crucian carp (*Carassius auratus*) [50], rainbow trout (*Oncorhynchus mykiss*) [51,52], and large yellow croaker (*Pseudosciaene crocea*) [53]. The results illustrated that curcumin could be added at the rate of 50 mg to 40 g/kg feed without harming the growth performance and health of the fish. Variation in the inclusion levels is probably associated with differences in feeding habits, fish size, duration, and farming conditions [43]. The enhanced growth performance may have resulted from the role of curcumin in enhancing the activity of digestive enzymes in the intestines [50,52]. In this regard, curcumin is known for its antibacterial activity against harmful intestinal microorganisms, thereby supporting beneficial microorganisms in their vital role in digestion and enhancing local intestinal immunity [19,20]. Curcumin has an attractive flavor as well, and may increase the palatability of feed and therefore feed intake [22]. Concurrently, the fish were able to utilize the feed more efficiently, leading to improved feed efficiency.

Intestinal immunity affects the entire body’s immunity (humoral and innate immunity) [54,55]. Improved local intestinal immunity is one of the potential roles of functional substances in aquafeed [56,57]. Curcumin has antibacterial activity, and may be involved in inhibiting the growth of harmful microorganisms [16]. In this regard, the results showed a lowered population of pathogenic bacteria (*Vibrio* spp. and *Faecal coliform*) in Gilthead seabream treated with curcumin, in a dose-dependent manner. These results have been attributed to the role of curcumin as a natural antibacterial supplement [58]. The enhanced growth performance in this study can probably be attributed to the antibacterial action of dietary curcumin against *Vibrio* spp. and *Faecal coliform* in the intestines of Gilthead seabream. However, further studies are required to understand the mode of action using microbiome techniques.

The measured hematological indices confirmed the beneficial role of curcumin on the health of Gilthead seabream [52]. Indeed, the detection of hematological features is a reliable tool to evaluate the impact of nutraceuticals on the health of fish [59]. High values of hematocrit (Hct) and hemoglobin (Hb) are diagnostic tools to assess the absence of anemic features [60]. The results showed increased Hb, Hct, RBCs, and WBCs in fish fed curcumin. This illustrated that curcumin has a beneficial effect on the metabolism and availability of nutrients in the blood of Gilthead seabream [57], as indicated by increased Hct and Hb values. The role of RBCs is to carry oxygen from the gills and transfer it to the body’s cells and tissues, to fulfill metabolic functions [61]. The results were concurrent with findings from Yonar et al. [52], who reported increased Hct, Hb, and RBCs in rainbow trout (*O. mykiss*) fed dietary curcumin supplements. The enhancement of RBCs, Hct, and Hb indicates efficient haemosynthesis and erythropoiesis, resulting from prevention of malnutrition and anemia [62]. The measurement of MCV is involved in detecting the average size of RBCs, while MCH refers to the amount of oxygen-carrying Hb inside RBCs. Further, high MCHC indicates that Hb is highly concentrated inside the RBCs [61]. In this study, the values of MCH, MCV, and MCHC showed insignificant differences among the groups, illustrating that the Gilthead seabream showed no anemic features induced by dietary curcumin. Similarly, rainbow trout fed dietary curcumin showed no changes in MCH, MCV, and MCHC comparing with the control [52]. In the present study, the levels of MCH, MCV, and MCHC recorded were 30.21 ± 0.39–32.90 ± 0.24, 99.58 ± 0.39–105.54 ± 0.30, and 30.17 ± 0.15–31.80 ± 0.09), respectively, while Yonar et al. [52] reported values of 42.13 ± 3.22–43.85 ± 4.51, 216.47 ± 15.27–225.62 ± 14.05, and 19.11 ± 3.10–20.03 ± 2.39 for MCH, MCV, and MCHC in rainbow trout, respectively. The differences between the values can probably be attributed to the differences in fish species, feeding habits, duration of curcumin feeding, and fish size.

The innate immune components in fish are the phagocytic cells (monocytes/macrophages and granulocytes) involved in resisting infection by pathogens [63]. Therefore, phagocytic activity is an essential response associated with activation of innate immunity to inhibit infection [64]. This study showed enhanced phagocytic activity in Gilthead seabream treated with curcumin. The results are in agreement with the findings from Yonar et al. [52], who reported enhanced phagocytic activity in rainbow trout fed dietary curcumin. Alongside increased phagocytic activity, the results showed an improved WBC count in Gilthead seabream fed dietary curcumin, indicating a robust immune response. Along the same lines, common carp [65] and rainbow trout [52] fed curcumin supplements displayed increased WBCs. The immunomodulating role of curcumin can probably be attributed to the activation of neutrophils and macrophages, to release reactive oxygen species [66]. Gao et al. [67] reported that dietary curcumin increased WBCs and phagocytic activity, leading to a higher expression of the cytokines involved in immunity.

Malnutrition and low feed quality induces oxidative stress, leading to impaired liver function and lipid metabolism [68]. This failure of the liver tissue results in irregular ALT, AST, and ALP values, while interrupted renal tissue can be expressed by a high urea level. The results showed no marked effects of curcumin on the biochemical blood metabolites of Gilthead seabream, indicating their healthy condition and lack of stress.

## 5. Conclusions

The impact of dietary curcumin on the performance of Gilthead seabream was investigated for the first time in this study. The results showed marked improvements in the growth performance, feed efficiency, and antibacterial capacity of the fish. Further, curcumin enhanced some hematological parameters in Gilthead seabream. Dietary curcumin is recommended at the level of 2–3% to improve the performance of Gilthead seabream.

## Figures and Tables

**Table 1:** Formulation and chemical composition of the basal diet.

Ingredient	%	Chemical Composition	%
Fish meal	40	Dry matter	90.74
Shrimp meal	5	Crude protein	44.20
Soybean meal	15	Ether extract	15.59
Yellow corn	10	Total ash	9.66
Corn gluten	6	Gross energy (Kcal/100 g)	516.70
Wheat middling	12		
Dicalcium phosphate	1		
Mineral and vitamin mixture	1		
Fish oil	10		
Total	100		

Vitamin mix (IU or mg/kg of diet): Vitamin D, 250,000 IU; Vitamin E, 4500 mg; Vitamin K_3_, 220 mg; Vitamin B_1_, 320 mg; Vitamin B_2_, 1090 mg; Vitamin B_5_, 2000 mg; Vitamin B_6_, 5000 mg; Vitamin B_12_, 116 mg; Pantothenate, 1000 mg; Folic acid, 165 mg; Choline, 60,000 mg; Biotin, 50 mg; Niacin, 2500 mg; and Vitamin C, 2000 mg; provided by Wuxi Hanove Animal Health Products Co., Ltd. (Jiangsu, China)., Mineral mix (g/kg of diet): CuSO_4_·5H_2_O, 2.5 g; FeSO_4_·7H_2_O, 28 g; ZnSO_4_·7H_2_O, 22 g; MnSO_4_·4H_2_O, 9 g; Na_2_SeO_3_, 0.045 g; KI, 0.026 g; and CoCl_2_·6H_2_O, 0.1 g; provided by Wuxi Hanove Animal Health Products Co., Ltd. (Jiangsu, China).

**Table 2:** Growth performance and feed utilization of Gilthead seabream fed varying levels of dietary curcumin for 150 days.

Item	Curcumin (%)				
	0	1.5	2	2.5	3
IBW (g)	20.18 ± 0.51	20.15 ± 0.54	20.04 ± 0.26	19.91 ± 0.12	19.74 ± 0.42
FBW (g)	112.56 ± 0.65 ^d^	115.20 ± 0.81 ^cd^	117.13 ± 0.93 ^bc^	119.30 ± 0.87 ^b^	126.11 ± 0.38 ^a^
WG (g)	92.38 ± 0.88 ^d^	95.06 ± 1.23 ^cd^	97.10 ± 0.96 ^bc^	99.39 ± 0.76 ^b^	106.36 ± 0.48 ^a^
SGR (%/day)	1.15 ± 0.02 ^b^	1.16 ± 0.02 ^b^	1.18 ± 0.01 ^b^	1.19 ± 0.00 ^b^	1.24 ± 0.01 ^a^
FCR	1.61 ± 0.02 ^b^	1.72 ± 0.01 ^a^	1.65 ± 0.01 ^b^	1.56 ± 0.03 ^c^	1.35 ± 0.01 ^d^
PER	1.41 ± 0.02 ^bc^	1.32 ± 0.01 ^c^	1.37 ± 0.01 ^c^	1.45 ± 0.03 ^b^	1.67 ± 0.01 ^a^
Survival (%)	91.11 ± 5.88	95.56 ± 4.44	97.78 ± 2.22	95.56 ± 4.44	100.00 ± 0.00

Values in the same row with different letters are significantly different (*p* < 0.05) (*n* = 3). IBW: initial body weight (g), FBW: final body weight (g), WG: weight gain (g), SGR: specific growth rate, FCR: feed conversion ratio, PER: protein efficiency ratio.

**Table 3:** Carcass composition and somatic indices of Gilthead seabream fed varying levels of dietary curcumin for 150 days.

Item	Curcumin (%)					
	Initial	0	1.5	2	2.5	3
Dry matter (%)	32.84 ± 1.28	33.17 ± 0.03	33.54 ± 0.44	32.68 ± 0.26	34.48 ± 0.36	33.23 ± 0.31
Crude protein (%)	57.28 ± 0.39	54.14 ± 0.14	53.90 ± 0.34	53.35 ± 0.38	53.55 ± 0.33	54.43 ± 0.24
Lipids (%)	25.78 ± 0.78	28.63± 0.25	28.45± 0.13	28.23 ± 0.14	28.39 ± 0.27	28.22 ± 0.55
Ash (%)	15.80 ± 0.15	15.94 ± 0.69	16.52 ± 0.20	17.16 ± 0.32	17.27 ± 0.15	16.74 ± 0.40
HSI (%)	-	2.18 ± 0.08	2.47 ^b^ ± 0.06	2.61 ± 0.17	2.70 ± 0.20	3.12 ± 0.07
VSI (%)	-	9.73 ± 0.39	10.02 ± 0.30	10.54 ± 0.22	9.78 ± 0.45	9.96 ± 0.31

HSI: hepatosomatic index; VSI: viscerasomatic index.

**Table 4:** Hematological parameters of Gilthead seabream fed varying levels of dietary curcumin for 150 days.

Item	Curcumin (%)				
	0	1.5	2	2.5	3
Hemoglobin (g/100 mL)	8.35 ± 0.55 ^c^	10.29 ± 0.30 ^b^	11.21 ± 0.65 ^ab^	12.16 ± 0.35 ^a^	12.54 ± 0.31 ^a^
RBCs (×10^6^/mm^3^)	3.56 ± 0.12 ^b^	3.64 ± 0.12 ^b^	4.20 ± 0.24 ^a^	4.46 ± 0.06 ^a^	4.63 ± 0.03 ^a^
Hematocrit (%)	31.05 ± 0.36 ^d^	33.20 ± 0.79 ^c^	37.81 ± 1.09 ^b^	39.78 ± 0.51 ^ab^	40.95 ± 0.17 ^a^
MCV (µm^3^/cell)	99.58 ± 0.39	102.96 ± 1.66	104.69 ± 0.44	105.29 ± 0.27	105.54 ± 0.30
MCH (pg/cell)	30.21 ± 0.39	31.41 ± 0.28	32.06 ± 0.14	32.26 ± 0.25	32.90 ± 0.24
MCHC (%)	30.17 ± 0.15	30.62 ± 0.17	31.38 ± 0.25	31.52 ± 0.37	31.80 ± 0.09
WBCs (×10^3^/mm^3^)	25.85 ± 89.75 ^d^	26.64 ± 150.89 ^c^	27.78 ± 116.93 ^b^	27.92 ± 33.48 ^b^	28.57± 136.68 ^a^
Lymphocyte (%)	40.88 ± 0.06	41.38 ± 0.51	41.72 ± 0.46	42.22 ± 0.29	42.53 ± 0.28
Monocyte (%)	4.44 ± 0.31	4.48 ± 0.32	4.87 ± 0.05	5.11 ± 0.17	5.35 ± 0.07
Eosinophil (%)	0.81 ± 0.04	0.84 ± 0.01	0.93 ± 0.02	1.71 ± 0.07	1.80 ± 0.04

Values in the same row with different letters are significantly different (*p* < 0.05) (*n* = 3). RBCs: red blood cells, MCV: mean corpuscular volume, MCH: mean corpuscular hemoglobin, MCHC: mean corpuscular hemoglobin concentration, WBCs: white blood cells.

**Table 5:** Blood biochemical indices of Gilthead seabream fed varying levels of dietary curcumin for 150 days.

Item	Curcumin (%)				
	0	1.5	2	2.5	3
ALT (U/I)	83.76 ± 0.12	84.49 ± 0.11	84.84c ± 0.03	85.51 ± 0.15	86.08 ± 0.26
AST (U/I)	81.33 ± 0.33	82.00 ± 0.00	82.33 ± 0.33	82.67 ± 0.33	83.00 ± 0.00
ALP (U/I)	91.30 ± 1.45	70.27 ± 0.66	69.55 ± 0.94	72.73 ± 1.11	61.30 ± 1.11
Albumin (g/dL)	3.23 ± 0.07	3.41 ± 0.08	3.65 ± 0.05	3.75 ± 0.06	3.83 ± 0.06
Urea (mg/dL)	4.53 ± 0.07	4.74 ± 0.05	4.76 ± 0.10	4.86 ± 0.04	5.44 ± 0.06
Total cholesterol (mg/dL)	971.00 ± 2.31	953.00 ± 4.93	940.00 ± 3.21	941.33 ± 1.45	959.00 ± 2.08

AST: aspartate aminotransferase; ALT: alanine aminotransferase; ALP: alkaline phosphatase.

## Data Availability

The datasets generated during and/or analysed during the current study are available from the corresponding author on reasonable request.
